# Physicochemical Properties of Water-Based Copolymer and Zeolite Composite Sustained-Release Membrane Materials

**DOI:** 10.3390/ma15238553

**Published:** 2022-12-01

**Authors:** Haonan Sun, Tao Lei, Jianxin Liu, Xianghong Guo, Jiangjian Lv

**Affiliations:** 1 College of Water Resource Science and Engineering, Taiyuan University of Technology, Taiyuan 030024, China; 2 College of Chemical Engineering and Technology, Taiyuan University of Technology, Taiyuan 030024, China

**Keywords:** membrane material, water-based copolymers, zeolite, physicochemical properties, coefficient of variation method

## Abstract

A nitrogen fertilizer slow-release membrane was proposed using polyvinyl alcohol (PVA), polyvinylpyrrolidone (PVP), epoxy resin, and zeolite as raw materials. The effects of the water-based copolymer (PVA:PVP) solution ratio A (A_1_–A_4_) and zeolite amount B (B_1_–B_4_) on the water absorption rate (*XS*), water permeability (*TS*), fertilizer permeability (*TF*), tensile strength (*KL*), elongation at break (*DSL*), and viscosity (*ND*) of the membrane were explored using the swelling method, a self-made device, and a universal testing machine. The optimal combination of the water-based copolymer and zeolite amount was determined by the coefficient-of-variation method. The results show that the effects of the decrease in A on *KL* and the increase in B on *KL* and *DSL* are promoted first and then inhibited. *DSL* and *ND* showed a negative response to the A decrease, whereas *XS*, *TS*, and *TF* showed a positive response. The effect of increasing B on *ND*, *TS*, and *TF* showed a zigzag fluctuation. In the condition of A_1_–A_3_, *XS* showed a negative response to the B increase, whereas in the condition of A_4_, *XS* was promoted first and then inhibited. Adding PVP and zeolite caused the hydroxyl stretching vibration peak of PVA at 3300 cm^−1^ to widen; the former caused the vibration peak to move to low frequencies, and the latter caused it to move to high frequencies. The XRD pattern shows that the highest peak of zeolite is located at 2θ = 7.18° and the crystallization peak of the composite membrane increases with the rise in the proportion of zeolite. Adding PVP made the surface of the membrane smooth and flat, and adding a small amount of zeolite improved the mechanical properties of the membrane and exhibited good compatibility with water-based copolymers. In the evaluation model of the physicochemical properties of sustained-release membrane materials, the weight of all indicators was in the following order: *TF* > *ND* > *TS* > *KL* > *XL* > *DSL*. The optimal membrane material for comprehensive performance was determined to be A_2_B_3_.

## 1. Introduction

With the extensive use of traditional fertilizers, economic issues [1], environmental issues [2], and food safety [3] are becoming increasingly obvious. Slow- and controlled-release fertilizers could reduce the nutrient release rate, greatly improve the utilization rate, and then reduce environmental pollution and economic loss [4,5]. The selection and preparation of membrane materials are keys to the performance of slow- and controlled-release fertilizers. High-quality membrane materials have the advantages of wide sources, low cost, and biodegradability [6,7,8]. In particular, zeolite has wide sources, low cost, good water retention capacity in soil, and a promoting effect on plant growth [9,10]. Polyvinyl alcohol (PVA) and polyvinylpyrrolidone (PVP) have good membrane formation and biodegradability [11,12]. An improved method for coupling multiple materials, including PVA and PVP chemical crosslinking, zeolite physical recombination to form a three-dimensional copolymerization network, and epoxy resin plasticization, was proposed in the present work.

The comprehensive performance of a single PVA membrane is not good. Water resistance could be improved via crosslinking with citrate [13], thermoplasticity could be improved via reacting with formaldehyde [14], and degradability could be improved via blending with SiO_2_ [15]. However, most of these methods focus on a single approach to modification, and the response of various indicators to modification is not consistent. For example, hyaluronic acid modification strategies could improve PVA hydrophilicity and biocompatibility but reduce mechanical properties [16]. The modification strategy of piperic acid could improve the tensile strength (*KL*) and water resistance of PVA but reduce its elongation at break (*DSL*) [17]. Membranes with good performance should have good degradation [18], mechanical properties [19], and other characteristics at the same time. The coupling modification method adopted in the present paper, which includes using PVA and PVP chemical crosslinking, zeolite physical recombination to form a three-dimensional copolymerization network, and epoxy resin plasticization, has not been reported. The effects of the group allocation ratio among PVA, PVP, and zeolite on the physical and chemical properties of membrane materials are still unclear and need to be further explored.

Research reports showed that the response strength and trend of each index of the membrane to the same factor are different, and the optimal treatment of each index lacks consistency. For example, adding the crosslinker glutaraldehyde at 0.3 mL brought Young’s modulus of the membrane to an optimal value of 30.94 MPa, whereas the *DSL* was reduced to a lower level of 16.27% [20]. With 0.5 mL glycerol, the *DSL* of the membrane reached the best value, and the *KL* reached the lowest level of 0.47 Mpa [21]. If the optimal treatment evaluation method based on a single index could have subjective one-sidedness, the multi-index objective comprehensive evaluation method based on the distribution weight–coefficient-of-variation method must be adopted. This method has been mainly applied in the fields of mechanical design and manufacturing [22], data envelopment analysis [23], and economic evaluation of wind power plants [24], proving reasonable feasibility. However, the application of a comprehensive evaluation of sustained-release membranes has not been reported and needs to be deeply researched.

This paper aimed to elucidate the effects of zeolite, the water-based copolymer ratio, and its coupling effect on the physicochemical properties of *XS*, *TS*, *TF*, *KL*, *DSL*, and *ND*. A comprehensive evaluation model of the physicochemical properties of membranes was constructed, and the optimal water-based copolymer–zeolite ratio was determined, providing a theoretical basis for the development of new environmentally friendly degradable membranes.

## 2. Materials and Methods

### 2.1. Materials

PVA 1799 (analytically pure) and PVP K30 (analytically pure) were purchased from Sinopharm Chemical Reagent Co., Ltd. (Shanghai, China). Epoxy resin (industrial grade) was purchased from China Shandong Yousuo Chemical Technology Co., Ltd. (Linyi, China). 4A zeolite (industrial grade) was purchased from China Shanxi Taiheng Technology Co., Ltd. (Jinzhong, China). Industrial 4A zeolite is a cage-connected structure consisting of 8 cubic octahedrons and 12 regular tetrahedrons formed by silicon-aluminum oxygen tetrahedral units, belonging to the cubic crystal system. The parameters of 4A zeolite were as follows: Specific surface area 510 m^2^/g; whiteness > 95%; apparent density, 0.3~0.5 g/cm^3^; average particle size, 2 μm.

### 2.2. Fabrication of Membrane

We weighed a certain amount of PVA and deionized water in a three-necked flask, which was in the oil bath pan. At the beginning of the experiment, the temperature of the oil bath pan was kept at 95 °C and the PVA was completely dissolved after approximately 1.5 h. Then, PVP was added when the solution was cooled to 60 °C, and the solution was stirred at a constant temperature until completely dissolved. Zeolite was added to the water-based copolymer solution for mixing and stirring for 1 h. Finally, the epoxy resin was added to the mixed solution and reacted for 2 h to obtain the membrane solution. We then extracted a certain amount of membrane solution and dried it in a calorstat to form a membrane.

### 2.3. Experimental Design

Four levels of A (PVA:PVP) were designated as A_1_–A_4_, with proportions of 8:0%, 7.3:0.7%, 6.6:1.4%, and 5.9:2.1%, respectively. Four levels of B were also designated as B_1_–B_4_ and the ratios in the solution were 0%, 0.25%, 0.5%, and 1%, respectively. The amount of epoxy resin added was 14 g, equal to 2% in the solution. A two-factor four-level comprehensive experimental design with 16 groups was adopted.

### 2.4. Experimental Methods

*XS* and *TS* were calculated by Formulas (1) and (2) in accordance with the methods of Han [25]. *TF* was calculated by Formula (3) in accordance with the method of Huang [26]. *KL* and *DLS* were calculated by Formulas (4) and (5) and measured using the C45 universal testing machine (Shenzhen Wanjian Test and Design Co., Ltd., Shenzhen, China). *ND* was measured using the NDJ-8S digital display rotational viscometer (Shanghai Lichen Bangxi Instrument Co., Ltd., Shanghai, China). The nitrogen release rate (K) of the membrane fertilizer was determined by the Bertalanffy model (Formula (6)).

(1) $$XS=\frac{M_{1}}{M_{2}}\times 100\%$$

(2) $$TS=\frac{\Delta M}{\mathrm{tS}}$$

(3) $$TF=\frac{C\times V}{C_{0}\times V}\times 100\%$$

(4) $$KL=\frac{F_{N}}{S}$$

(5) $$DLS=\frac{L_{a}-L_{0}}{L_{0}}\times 100\%$$

(6) $$N=N_{0}{\left(1-{\mathrm{be}}^{-\mathrm{kt}}\right)}^{3}$$

where *XS* is the water absorption of the membrane (%), M_1_ is the saturation mass of the membrane material, M_2_ is the original quality of membrane material, *TS* is the water permeability of the membrane, ΔM is the mass change of silica gel (g), t is the water absorption time of the membrane (h), S is the permeable area of the membrane (cm^2^), *TF* is the fertilizer permeability of the membrane (%), C_0_ is the initial concentration of urea (g/L), V is the volume of distilled water and the volume of urea solution (L), C is the urea concentration (g/L), *KL* is the tensile strength of the sample (MPa), F_N_ indicates the maximum tension that the sample could withstand (N), S is the cross-sectional area of the sample (m2), *DLS* is the elongation at break of the sample (%), L_a_ indicates the length of the sample before breaking (cm), L_0_ is the length of the sample after breakage (cm), N is the cumulative release rate of urea, t is the release time, and K is the nitrogen release rate.

### 2.5. Data Processing and Statistical Analysis

IBM SPSS Statistics 22 data analysis software was used for the analysis of variance. Microsoft Office 2019 was used for data processing and table drawing. Origin2019b was used for graphical drawing. SPSSPRO 1.1.7 was used to determine the index weight and construct the comprehensive evaluation model of the membrane material.

## 3. Results and Analysis

### 3.1. Effect of Water-Based Copolymer Ratio and Zeolite Amount on the Hydrophilicity of Membrane Materials

**Figure 1:**
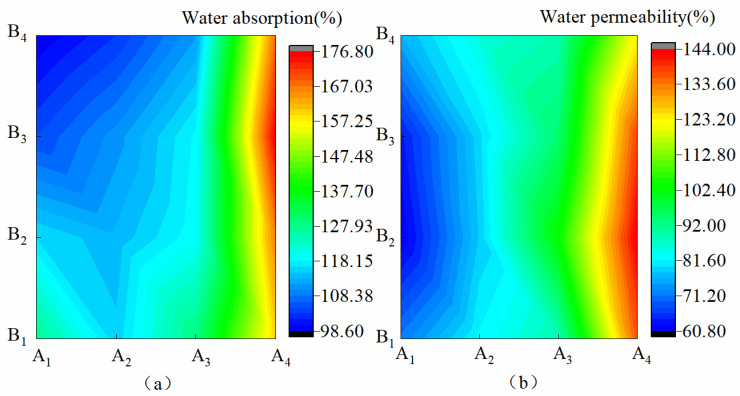
Hydrophilicity of membrane materials under different ratios of water-based copolymer and zeolite amounts. (**a**) Water absorption, (**b**) Water permeability.

Figure 1 shows the hydrophilic characteristics of the membrane materials under different water-based copolymer ratios and zeolite amounts. Figure 1a shows that in the condition of A_1_–A_3_, *XS* was monotonically decreased by 22.64%, 10.32%, and 17.22% with the increase in B, respectively. *XS* presented a negative response to the B increase. In the A_4_ condition, when B increased from B_1_ to B_3_, *XS* increased by 10.4%; when B increased from B_3_ to B_4_, *XS* decreased by 5.0%. This finding showed that the increase in B on *XS* was first promoted and then inhibited. As shown in Figure 1a, *XS* produced jagged fluctuations when A decreased, but the overall *XS* with the A decrease presented a positive response. A, B, and A*B exhibited a significant effect on *XS* (*p* ˂ 0.01), and the size of the influence was as follows: A > A*B > A. Figure 1b shows a slight fluctuation in *TS* when B was increased, and the effect of such an increase on *TS* was not significant (*p* > 0.05). In the condition of B_1_–B_4_, when A decreased from A_1_ to A_4_, *TS* significantly increased by 90.02%, 136.24%, 116.56%, and 61.56%, respectively. Thus, the decrease in A showed a significant positive response on *TS* (*p* ˂ 0.01). The response of *TS* to A was the most significant at B_2_. The influence of A, B, and A*B on *TS* was in the order A > A*B > B.

### 3.2. Effect of Water-Based Copolymer Ratio and Zeolite Amount on the Mechanical Properties of Membrane Materials

**Figure 2:**
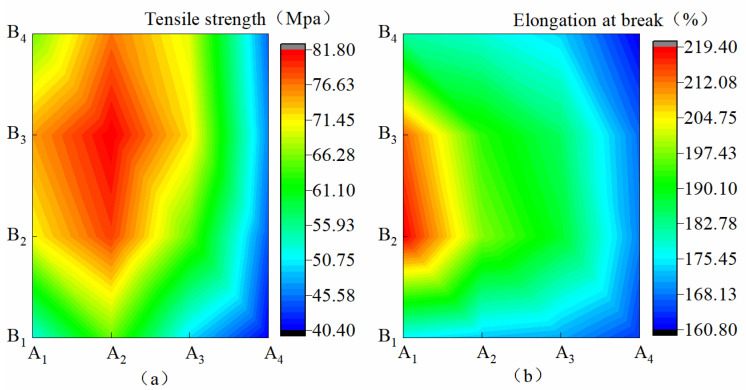
Mechanical properties of membrane materials under different ratios of water-based copolymer and zeolite amounts. (**a**)Tensile strength, (**b**) Elongation at break.

Figure 2a shows that when B increased from B_1_ to B_3_, *KL* increased by 13.72–43.31%, and when B increased from B_3_ to B_4_, *KL* decreased by 3.31–12.22%. This finding showed that the increase in B on *KL* was first promoted and then suppressed. When A decreased from A_1_ to A_2_, *KL* increased by 8.97–25.16%, and when A decreased from A_2_ to A_4_, *KL* decreased by 38.03–44.21%. This finding showed that the decrease in A on *KL* was first promoted and then suppressed. Figure 2b shows that when B increased from B_1_ to B_2_ and from B_2_ to B_4_, *DLS* increased by 2.75–44.74% and 4.64%–17.26%, respectively. This finding revealed that the increase in B on *DLS* was first promoted and then inhibited. In the condition of B_1_–B_4_, when A decreased from A_1_ to A_4_, *DLS* significantly increased by 6.72%, 23.08%, 21.58%, and 11.34%, respectively. The inhibitory effect was the most obvious under the B_2_ condition. A, B, and A*B all showed a significant effect on *KL* (*p* ˂ 0.01), and B had a significant effect on *DLS* (*p* < 0.05). On the contrary, A and A*B had no significant effect on *DLS*. The influences of A, B, and A*B on *KL* and *DLS* were A > B > A*B and B > A > A*B, respectively.

### 3.3. Effect of Water-Based Copolymer Ratio and Zeolite Amount on the Viscosity of Membrane Materials

**Figure 3:**
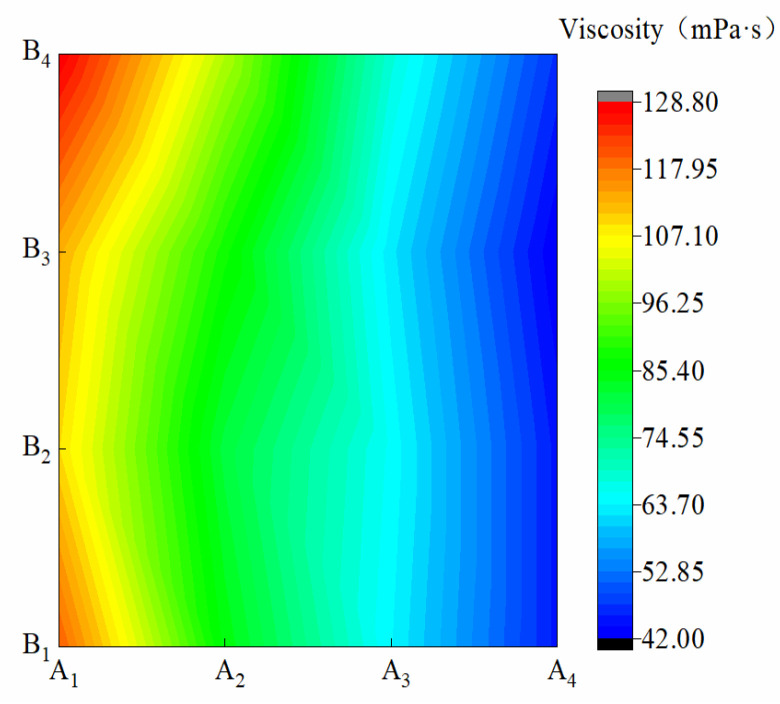
Viscosity of membrane materials under different ratios of water-based copolymer and zeolite amounts.

Figure 3 shows the viscosity of membrane materials under different water-based copolymer ratios and zeolite amounts. In the condition of A_1_–A_4_, the *ND* variations caused by B were 5.52%, 6.16%, 2.55%, and 3.08%, respectively, suggesting that the B increase had less effect on *ND* with the water-based copolymer at the same level. In the condition of B_1_–B_4_, when A decreased from A_1_ to A_4_, *ND* significantly decreased by 81.8, 75, 68, and 67 mPa·s, respectively. This finding showed that the A decrease inhibited *ND*, and this inhibition was most obvious under the B_1_ condition. A, B, and A*B had a significant effect on *ND* (*p* ˂ 0.01), and their influence on *TS* was in the following order: A > B > A*B.

### 3.4. Effect of Water-Based Copolymer Ratio and Zeolite Amount on the Fertilizer Permeability of Membrane Materials

**Figure 4:**
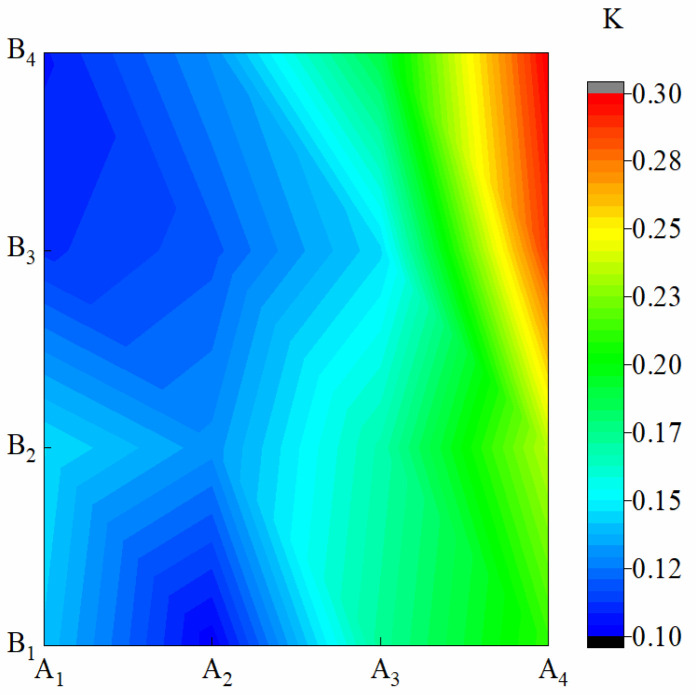
Release rate of membrane materials under different ratios of water-based copolymer and zeolite amounts.

Figure 4 shows the K of membrane materials under different water-based copolymer ratios and zeolite amounts. In the condition of A_1_–A_3_, the amplitude of variations of K caused by B were 13.67%, 15.60%, and 7.27%, respectively, and a jagged fluctuation in K was observed. Under the A_4_ condition, when B increased from B_2_ to B_3_, K increased by 18.64%. This finding showed that when the proportion of PVA in A dropped to a certain extent, the addition of B could have a greater effect on the sustained-release performance of the membrane. In the condition of B_1_–B_4_, when A decreased from A_1_ to A_4_, K monotonically increased by 33.60%, 38.08%, 61.79%, and 64.54%, respectively. K presented a significant negative response to the A decrease (*p* ˂ 0.01). B and A*B also showed a significant effect on *TF* (*p* ˂ 0.01). The influence of A, B, and A*B on *TF* was as follows: A > A*B > B.

### 3.5. Infrared Spectroscopic Analysis

**Figure 5:**
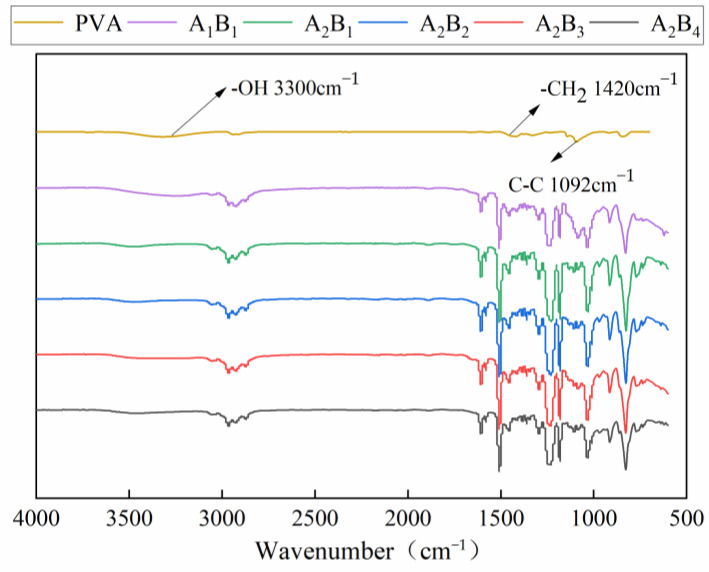
IR spectra of membrane materials under different ratios.

Figure 5 shows the infrared spectra of the membrane material under different water-based copolymer ratios and zeolite amounts. The typical peaks in the PVA spectrum mainly include the stretching vibration absorption peak of the -OH bond near 3300 cm^−1^ [27], the bending vibration peak of the -CH_2_ bond at 1420 cm^−1^, and the vibration absorption peak of the C-C skeleton at 1092 cm^−1^ [28]. A comparison of the spectra of A_1_B_1_ and PVA showed that the hydroxyl stretching vibration peak at 3300 cm^−1^ in the infrared spectrum of the membrane material widened and moved to the direction of a low wave number after adding PVP and epoxy resin. The bending vibration peak of the -CH_2_ bond at 1420 cm^−1^ and the vibration absorption peak of the C-C skeleton at 1092 cm^−1^ of PVA were considerably enhanced, indicating that more excited electrons were generated in the atoms of the membrane material and the absorption of infrared light was significantly enhanced. The formation of a hydrogen bond could weaken the -OH bond, resulting in the reduction in -OH bond vibration frequency, which is a typical athochromic-shift phenomenon [29]. A comparison of the spectra of A_1_B_1_ and A_2_B_1_ revealed that the stretching vibration peak of the -OH bond further widened and extended to the low band after the addition of PVP, and the peak intensity showed an enhancement trend. A_2_B_1_, A_2_B_2_, A_2_B_3_, and A_2_B_4_ had obvious absorption peaks in the 2500–3000 cm^−1^ band, indicating that the complexation reaction occurred in PVA and PVP molecules through positive and negative charge attraction, and the two molecules formed intermolecular hydrogen bonds.

### 3.6. X-Ray Diffractive Analysis

**Figure 6:**
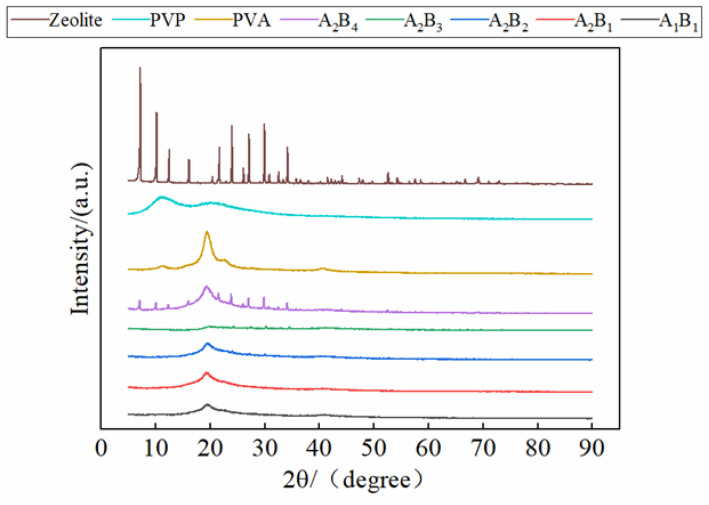
XRD of membrane materials under different ratios.

Figure 6 shows the XRD pattern of membrane materials under different water-based copolymer and zeolite amounts. It can be seen from Figure 6 that the main characteristic peaks of zeolite are 2θ = 7.18°, 9.52°, 16.24°, 26.18°, which belong to the crystal plane of (2 0 0), (3 0 0), (2 2 1), (2 1 4) of 4A zeolite, respectively, and the strongest diffraction peak of (2 0 0) crystal plane appears at 2θ = 7.18°. PVA has a typical crystallization peak at 2θ = 19.35°. The composite membrane also has a typical crystallization peak at 2θ = 19.35°, because PVA is the main component of the composite membrane. Comparing the XRD diffractograms of the zeolite with the zeolite-modified water-based copolymer composite membranes, some crystalline peaks related to the zeolite disappeared in the composite membranes, and the intensity of the crystalline peaks was lower in the composite membranes than in the zeolite. This indicates that some reactions occurred between the zeolite and the water-based copolymer. Comparing the typical peaks of composite membranes, it can be seen that with the increase in the proportion of zeolite in the composite membrane, the typical peaks show a rising trend. This indicates that zeolite has an obvious modification effect on composite membranes.

### 3.7. Effect of Water-Based Copolymer Ratio and Zeolite Amount on the Surface Property of Membrane Materials

**Figure 7:**
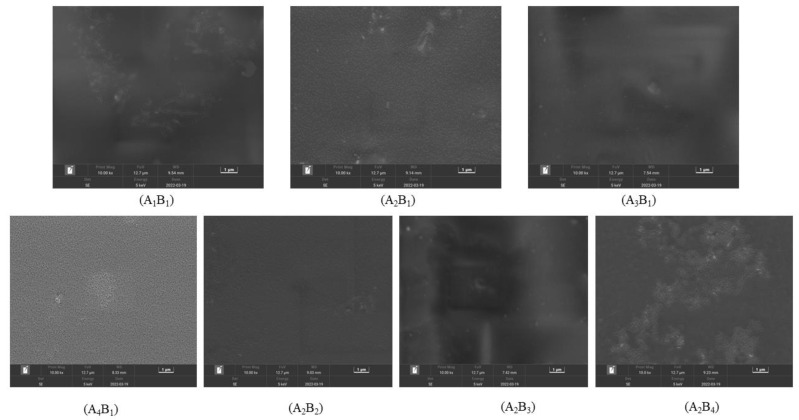
Electron microscopic photo of the membrane material surface.

Figure 7 shows the SEM photos of the membrane surface structure. Without the addition of PVP, the surface of A_1_B_1_ increased and cracked. After PVP was added, the surfaces of A_2_B_1_, A_3_B_1_, and A_4_B_1_ became flat and smooth, and the cracks disappeared. However, with the increase in the PVP ratio, a slight agglomeration phenomenon occurred on the membrane surface, which may be related to the reaction temperature and time of the test. When the addition amount of B was 0, the surface of A_2_B_1_ was smooth and flat, indicating that PVA and PVP were well combined and compact. When the B addition amount was 1.75 g, the surface of A_2_B_2_ was also smooth and flat, indicating that a small amount of B addition exhibited good compatibility with A. With the continuous addition of B, the surface flatness and uniformity of A_2_B_3_ and A_2_B_4_ worsened, and the surface of the membrane material increased. These findings showed that the ratio of A_2_ and B_2_ could obtain a membrane with excellent surface properties.

### 3.8. Comprehensive Evaluation

**Figure 8:**
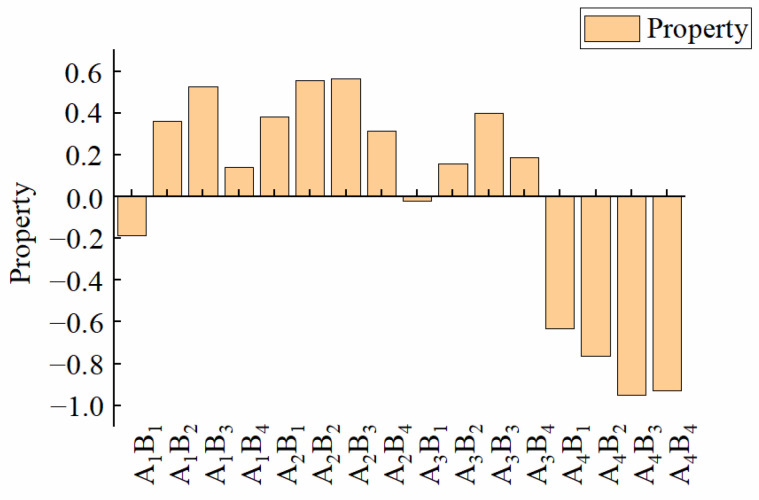
Comprehensive score for different treatments.

The previous analysis showed that A and B had different effects on *TF*, *ND*, *TS*, *KL*, *XL*, and *DSL*. The results of the optimal water-based copolymer–zeolite amount combination based on a single indicator were not consistent. Objectively and comprehensively selecting the optimal treatment of comprehensive performance was found to be difficult, so a comprehensive evaluation of various indicators of the membrane materials must be conducted. First, the coefficient-of-variation method was used to determine the weight coefficients of various indicators, and the results are shown in Table 1. The weight of each indicator was in the following order: *TF* > *ND* > *TS* > *KL* > *XL* > *DSL*. *TF* had the largest weighting proportion (24.20%), whereas *DSL* had the smallest (6.06%). This finding showed that *TF* was the most important index of the membrane material, and *DSL* was a relatively minor indicator. By establishing the weights, typical indicators that reflect the properties of the membrane could be selected. It also helps build a more reasonable model evaluation system and obtain more reasonable evaluation results. Then, the Z-score standardization method was used to standardize the data of each indicator. Finally, the standardized data were combined with the weight coefficients to obtain the comprehensive score of the membrane, and the result is shown in Figure 8. The comprehensive score could be used as an objective evaluation index to judge the quality of membrane materials. The results showed that A_2_B_3_ had the best comprehensive performance, with a score of 0.57, whereas A_4_B_3_ had the worst comprehensive performance, with a score of −0.95.

## 4. Discussion

### 4.1. Properties of Membrane Materials

*XS* is an important index for evaluating the properties of membrane materials. Studies have shown that the lower the *XS* is, the better the sustained-release performance of the membrane [30]. The results in the present paper showed that adding zeolite could inhibit *XS*, and this inhibition effect was most obvious at the A_2_ level of the water-based copolymer. This finding is mainly related to the internal configuration of zeolite [31]. Previous studies have found that the addition of biochar modification to water-based copolymers achieved good results [32]. Similar to biochar [33], the porous zeolite used in the present paper has a unique “tetrahedral” structure [34], physical adsorption, and ion exchange characteristics [35]. It blends with polymers to form three-dimensional network structures and change the properties of membrane materials.

The results of this study showed that the K values varied under different treatments, but the sustained-release performance reached the optimum under the A_2_B_1_ treatment. This finding is mainly closely related to the chemical crosslinking density and compatibility between materials [36]. As shown in Figure 6, the surface of A_2_B_1_ was smooth. Under this treatment, the compatibility between PVA and PVP was the best, and the chemical crosslinking density between materials was the largest. So, the diffusion rate of water and urea molecules through the crosslinked polymer was low [37,38,39]. With the formation of hydrogen bonds between the carbonyl group of PVP and the hydroxyl group of PVA, the -OH stretching vibration peak shifted. The results of the present study also showed that the nutrient release curve of the membrane material was rapid in the initial stage, and the release rate slowed down after reaching the inflection point at a certain period. This phenomenon is due to the fast membrane material initial water swelling of urea molecules through the polymer network. After a certain period, the membrane gradually expanded to saturation, and the diffusion channel became narrow. The permeability coefficient of water and urea molecules through the polymer membrane decreased, and the rate of fertilizer penetration slowed down [40].

By constructing the model, Hennepe [41] concluded that zeolite had a significant effect on polymer properties. In the present paper, the effect of zeolite addition on the mechanical properties of the membrane was shown to be promoted first and then inhibited. Chang [42] found that the increase in zeolite content did not significantly improve the mechanical properties of the material. The *DLS* of UHMWPE material increased first and was then inhibited, whereas the *KL* gradually decreased. The membrane materials constructed in the present paper could form a three-dimensional network structure, which could help reduce the twisting of molecular chains [43]. An appropriate increase in zeolite could improve the membrane’s crosslinking density. As the number of nodes per unit volume in the network structure increased, the free volume in the polymer decreased. The molecular chain torsion was reduced, and the *KL* of the membrane increased [44]. However, when excessive zeolite was added, the free volume of the water-based copolymer decreased continuously, and the addition of zeolite destroyed the original crosslinking node, thus leading to decreased *KL*.

### 4.2. Optimization of Evaluation Method

In the comprehensive evaluation system, the establishment of reasonable weights is crucial to whether the decision-making problem could be solved, and it is a key factor in evaluation accuracy [45]. Zou [46] concluded that the mechanical properties and water resistance of the composite membrane reached the optimum when the additional amount of KGM was 0.3%. If the evaluation method, which was based on the mechanical properties and water resistance indices, reached the optimum and was adopted in this paper, the optimal treatment would be A_1_B_2_, different from the result after the index weight was reasonably established in this paper. The reason for the above difference is that the importance degree of indicators differed, and the corresponding optimal treatment of different indicators was inconsistent. Selecting the optimal treatment by only using some indicators to achieve the optimum was difficult. Although A_1_B_2_ had excellent mechanical properties and water resistance, its *ND* and *TF* indicators were poor. Thus, it could not be used as an optimal treatment. In this paper, the coefficient-of-variation method was used to determine the importance of the membrane material indicators. In the membrane material score calculation of comprehensive consideration of all indicators, the results could be more objective and reasonable.

Previous research used principal component analysis [47] to determine the optimal membrane ratio, providing theoretical guidance for the optimization of membrane material and the ratio between components. The principal components extracted by a principal component analysis were at least two, and the cumulative contribution rate of variance was more than 85%, which could better reflect the original variable information [48,49]. When a principal component analysis was used for a comprehensive evaluation in the present paper, the extraction criteria with an eigenvalue greater than 1 [50] were adopted, and only one principal component with a variance contribution rate of 80.179% was extracted (Table 2). Principal component analysis was used to determine that a principal component with a low variance contribution rate does not better reflect the original variable information. It could not specifically obtain the weight of each index, and partial one-sidedness could occur. In this paper, the coefficient of variation and standard deviation of the indicators were obtained using the coefficient-of-variation method, and the weights of all the indicators were determined. The method could objectively and accurately describe the original variable information (Table 1). Therefore, the reasonable choice of method in comprehensive evaluation is conducive to building a more reasonable model evaluation system and obtaining more reasonable evaluation results.

## 5. Conclusions

The effects of the A decrease on *KL* and the B increase on *KL* and *DSL* were promoted first and then inhibited. *DSL* and *ND* showed a negative response to the A decrease, whereas *XS*, *TS*, and *TF* showed a positive response. The effect of increasing B on *ND*, *TS*, and *TF* showed a zigzag fluctuation. Under the condition of A_1_–A_3_, *XS* showed a negative response to the B increase, and under the A_4_ condition, *XS* was promoted first and then inhibited. The addition of PVP and zeolite could cause the hydroxyl expansion vibration peak of PVA at 3300 cm^−1^ to widen and move. The XRD pattern shows that the highest peak of zeolite is located at 2θ = 7.18°, and the crystallization peak of the composite membrane increases with the rise of the proportion of zeolite. The addition of PVP made the surface of the membrane flat and smooth. The addition of a small amount of zeolite improved the mechanical properties of the membrane and exhibited good compatibility with water-based copolymers. Meanwhile, adding excessive zeolite could lead to poor flatness and smoothness of the membrane. The influence of different factors on the *XS*, *TS*, and *TF* of the membrane materials was as follows: A > A*B > B; the influence on the *KL* and *ND* of the membrane materials was as follows: A > B > A*B; and the influence on the *DSL* was B > A > A*B. The weight of all indicators was in the order *TF* > *ND* > *TS* > *KL* > *XL* > *DSL*. The best performance was found in the A_2_B_3_ treatment, and the worst was in the A_4_B_3_ treatment.

## Figures and Tables

**Table 1:** Weight coefficient of different indices.

Index	Average Value	Standard Deviation	CV Value	Weight (%)
Water absorption	127.52	25.39	0.20	13.00
Water permeability	94.62	26.71	0.28	18.44
Viscosity	78.93	28.64	0.36	23.71
Fertility permeability	0.17	0.06	0.37	24.20
Tensile strength	62.46	13.94	0.22	14.58
Elongation at break	181.97	16.88	0.09	6.06

**Table 2:** Eigenvalues and cumulative variance contribution rates of membrane evaluation factors.

Ingredient	Eigenvalues	Variance Contribution Rate (%)	Cumulative Variance Contribution Rate (%)
1	4.811	80.179	80.179
2	0.557	9.285	89.464
3	0.430	7.165	96.629
4	0.134	2.237	98.866
5	0.068	1.134	100.00
6	0.000	0.000	100.00

## Data Availability

Not applicable.
